# Escore para Avaliação de Prognóstico em Pacientes com Endocardite Infecciosa

**DOI:** 10.36660/abc.20230814

**Published:** 2024-08-06

**Authors:** Alfredo José Mansur

**Affiliations:** 1 Instituto do Coração Faculdade de Medicina Universidade de São Paulo São Paulo SP Brasil Instituto do Coração - Faculdade de Medicina da Universidade de São Paulo - Cardiologia Clínica, São Paulo, SP – Brasil

**Keywords:** Prognóstico, Endocardite Bacteriana, Pacientes Internados, Registros Eletrônicos de Saúde

Critérios estruturados de estimativa prognóstica são uma questão chave no tratamento de pacientes e podem ser uma ajuda adicional para guiar o aconselhamento adequado dos pacientes, bem como dar suporte às decisões terapêuticas aos médicos responsáveis pelos pacientes.^1^ A avaliação prognóstica também pode ser expressa como escores de risco; muitos deles foram desenvolvidos para casuísticas de estudos populacionais epidemiológicos^2-4^ou grupo específico de pacientes como pacientes em terapia intensiva^5-7^ com doença cardiovascular suspeita ou ativa^2,8,9^ ou uma doença específica como endocardite infecciosa.^10^

Na presente publicação^11^os autores avaliaram estimativas de prognóstico intra-hospitalar e pós alta hospitalar com o emprego do escore “Sharpen” em comparação ao índice de comorbidade de Charlson em 168 pacientes (179 admissões) com endocardite infecciosa durante dezesseis anos (2000-2016) baseados na revisão de prontuários hospitalares. Sharpen é um acrônimo de sete condições clínicas (expressas em inglês) (Pressão arterial Sistólica, Insuficiência Cardíaca, Idade, Função renal, Pneumonia, Taxa elevada de proteína C reativa e Não uso de drogas intravenosas – SHARPEN) submetido a análise multivariada (regressão logística) convertendo β coeficientes em pesos.^12^

Na casuística que se publica neste fascículo^11^ o *Staphylococcus aureus* foi o microrganismo mais frequente (86%). Sessenta e oito pacientes foram submetidos a cirurgia devido a insuficiência cardíaca aguda índice (79,4%) ou infecção descontrolada (41,2%). Eles concluíram que o escore Sharpen teve uma performance apropriada em relação ao índice de comorbidade de Charlson e talvez uma melhor acurácia (72,1, faixa 59,8-82,3 vs. 62, faixa 54,5-69,1) em pacientes não operados, apesar de alguma sobreposição das faixas de acurácia.

Interessantemente, condições clínicas que foram precursoras de complicações, eram as variáveis do escore estudado, bem como outros estudos de prognóstico.^10^ Em uma perspectiva clínica, idade, sexo, infecção por microrganismo e condições cardíacas e comorbidades, não devem faltar na estimativa de risco independentemente de um valor de coeficiente; os números nem sempre traduzem informações clínicas relevantes sem uma fase de validação. A presença de complicações tem sido reconhecida há muito tempo como índice prognóstico desfavorável, principalmente na endocardite por *Staphyloccocus aureus*^13^ e outras variáveis clínicas como infecções por microrganismos e cirurgia.^10^ A cirurgia às vezes pode modificar a proporcionalidade dos riscos durante o período de tratamento, fazendo com que algumas premissas, falhas para o modelo estatístico baseado em riscos proporcionais, possam não atender os pressupostos da modelagem estatística.^14,15^

Endocardite é uma doença rara e cada caso pode apresentar características muito individuais que podem dificultar a translação da análise de casuística de grupo, geralmente retrospectiva, para a prática clínica de decisões individuais.^15,16^ Para os médicos, a supervisão clínica cuidadosa de cada paciente, o diagnóstico no tempo apropriado da condição clínica e das complicações, contribuem para o sucesso das terapias médicas e cirúrgicas em pacientes com endocardite infecciosa, incluindo aqueles de alto risco.^17^
