# Supplementary material for: Escore para Avaliação de Prognóstico em Pacientes com Endocardite Infecciosa
Source: Arq Bras Cardiol. 2024 Aug 6;120(12):e20230814. [Article in Portuguese] doi: 10.36660/abc.20230814 (PMC11364443; doi:10.36660/abc.20230814)
Supplement: Translation

# Translation: Score For Prognosis Assessment In Patients With Infective Endocarditis

**Authors:** Alfredo José Mansur

**Affiliations:** 1 Brazil Instituto do Coração - Faculdade de Medicina da Universidade de São Paulo - Cardiologia Clínica, São Paulo, SP - Brazil

**Keywords:** Prognosis, Endocarditis,Bacterial, Inpatients, Electronic Health Records

Structured prognostic estimation criteria are a key issue in patient treatment and can be an additional help to guide appropriate patient counseling, as well as support therapeutic decisions for doctors responsible for patients.^1^ Prognostic assessment can also be expressed as risk scores; many of them were developed for case series of epidemiological population studies^2-4^ or a specific group of patients such as patients in intensive care^5-7^ with suspected or active cardiovascular disease^2,8,9^ or a specific disease such as infective endocarditis.^10^

In the present publication,^11^ the authors evaluated in-hospital and post-discharge prognosis estimates using the “Sharpen” score in comparison to the Charlson comorbidity index in 168 patients (179 admissions) with infective endocarditis over sixteen years (2000-2016) based on the review of hospital records. Sharpen is an acronym for seven clinical conditions (Systolic Blood Pressure, Heart Failure, Age, Renal Function, Pneumonia, Elevated C-reactive protein Rate, and Non-Use of Intravenous Drugs – SHARPEN) subjected to multivariate analysis (logistic regression) converting β coefficients into weights.^12^

In the series published in this issue,^11^ *Staphylococcus aureus* was the most common microorganism (86%). Sixty-eight patients underwent surgery due to index acute heart failure (79.4%) or uncontrolled infection (41.2%). They concluded that the Sharpen score had an appropriate performance in relation to the Charlson comorbidity index and perhaps better accuracy (72.1, range 59.8-82.3 vs. 62, range 54.5-69.1) in non-operated patients, despite some overlap in the ranges of accuracy.

Interestingly, clinical conditions that were precursors to complications were the variables in the score studied, as well as other prognostic studies.^10^ From a clinical perspective, age, sex, microorganism infection and cardiac conditions and comorbidities should not be missing from the risk estimate regardless of a coefficient value; numbers do not always translate relevant clinical information without a validation phase. The presence of complications has long been recognized as an unfavorable prognostic index, especially in endocarditis caused by *Staphylococcus aureus*^13^ and other clinical variables such as infections caused by microorganisms and surgery.^10^ Surgery can sometimes modify the proportionality of risks during the treatment period, meaning that some assumptions, flaws for the statistical model based on proportional risks, may not meet the assumptions of statistical modeling.^14,15^

Endocarditis is a rare disease, and each case can present very individual characteristics that can make it difficult to translate group case analysis, generally retrospective, into the clinical practice of individual decisions.^15,16^ For doctors, careful clinical supervision of each patient and timely diagnosis of the clinical condition and complications contribute to the success of medical and surgical therapies in patients with infective endocarditis, including those at high risk.^17^
